# Simultaneous Monitoring of Soil Water Content and Salinity with a Low-Cost Capacitance-Resistance Probe

**DOI:** 10.3390/s121217588

**Published:** 2012-12-18

**Authors:** Elia Scudiero, Antonio Berti, Pietro Teatini, Francesco Morari

**Affiliations:** 1 Department of Agronomy, Food, Natural Resources, Animals and Environment (DAFNAE), University of Padua, Viale dell’Università, 16, 35020 Legnaro, Italy; E-Mails: elia.scudiero@studenti.unipd.it (E.S.); antonio.berti@unipd.it (A.B.); 2 Department of Civil, Environmental and Architectural Engineering (ICEA), University of Padua, Via Trieste, 63, 35131 Padova, Italy; E-Mail: teatini@dmsa.unipd.it

**Keywords:** capacitance-resistance probe, salinity, water content, pore-water electrical conductivity, probe calibration

## Abstract

Capacitance and resistivity sensors can be used to continuously monitor soil volumetric water content (*θ*) and pore-water electrical conductivity (*EC_p_*) with non-destructive methods. However, dielectric readings of capacitance sensors operating at low frequencies are normally biased by high soil electrical conductivity. A procedure to calibrate capacitance-resistance probes in saline conditions was implemented in contrasting soils. A low-cost capacitance-resistance probe (ECH2O-5TE, 70 MHz, Decagon Devices, Pullman, WA, USA) was used in five soils at four water contents (*i.e.*, from dry conditions to saturation) and four salinity levels of the wetting solution (0, 5, 10, and 15 dS·m^−1^). *θ* was accurately predicted as a function of the dielectric constant, apparent electrical conductivity (*EC_a_*), texture and organic carbon content, even in high salinity conditions. Four models to estimate pore-water electrical conductivity were tested and a set of empirical predicting functions were identified to estimate the model parameters based on easily available soil properties (e.g., texture, soil organic matter). The four models were reformulated to estimate *EC_p_* as a function of *EC_a_*, dielectric readings, and soil characteristics, improving their performances with respect to the original model formulation. Low-cost capacitance-resistance probes, if properly calibrated, can be effectively used to monitor water and solute dynamics in saline soils.

## 1. Introduction

Coastal farmlands are often threatened by saltwater contamination that poses a serious risk for drinking water quality and agricultural activities. To control and evaluate the hazard of soil salinity, accurate measurements of soil water content and solute concentrations are needed. The term salinity refers to the presence of the major dissolved inorganic solutes (basically Na^+^, Mg^2+^, Ca^2+^, K^+^, Cl^−^, SO_4_^2−^, HCO_3_^−^, NO_3_^−^, and CO_3_^2−^ ions) in the soil [1]. The salinity of a solution can be quantified in terms of its electrical conductivity (EC; dS·m^−1^), which is strictly related to the total concentration of dissolved salts, with 1 dS m^−1^ being approximately equivalent to 10 meq·L^−1^ at 25 °C [2]. Soil salinity is generally determined by measuring the electrical conductivity of aqueous extracts of saturated soil-pastes (*EC_e_*) or of other soil to water ratio extracts. However, such methods of investigation are destructive, time-consuming, and usually not representative of the real salinity status of soils in field conditions [1]. To determine the real (*i.e.*, at actual soil water contents) stress conditions affecting crops and to monitor fluxes of salts (e.g., upward fluxes in the vadose zone) the electrical conductivity of the pore-water (*EC_p_*) should be measured instead. Multi-sensor probes have recently been developed in order to assess water content and electrical conductivity with continuous and non-destructive measurements.

## 2. Methodological Issues

### 2.1. Water Content Measurements

The capacitance (dielectric) technique has been widely used to estimate soil volumetric water content (*θ*) [3]. Capacitance sensors induce an alternating electric field in the surrounding medium. The total complex impedance is obtained by quantifying the voltage and the current induced by the electric field on the sensor electrodes. The impedance is related to the complex permittivity (or dielectric constant; *ε_r_*) of the surrounding medium. The volume of the induced electric field depends mainly on the size and shape of the sensor electrodes. Moreover, the electric field decays rapidly, being inversely proportional to the square of the distance. Topp *et al.*[4] noticed a strict correlation between *ε_r_* measured by time domain reflectometry (TDR) and soil water content. They therefore proposed an empirical third-degree polynomial in *ε_r_* to calculate *θ*. The complex permittivity of the soil measured by dielectric sensors is the sum of soil real (*ε’*) and imaginary (*ε″*) permittivity (dielectric loss):

(1) $$\varepsilon_{r}=\varepsilon^{\prime }-j\times \varepsilon^{\prime \prime }$$

where *j*^2^ = −1. The value of *θ* is related to *ε’* only. On the other hand, *ε″* changes according to soil salinity, soil temperature (*T*), and the operating frequency of the sensor [5–10]. Especially in low-cost sensors working at low frequencies (<1 GHz), the contribution of *ε″* in saline soils cannot be ignored [3,11,12]. It is therefore essential to consider the influence of *ε″* in *ε_r_* measurements in order to gain correct *θ* estimations.

### 2.2. Pore-Water Electrical Conductivity Assessment

The determination of the pore-water electrical conductivity is a difficult task as it cannot be directly related to any sensor output. Typically sensors measure soil bulk (or apparent) electrical conductivity (*EC_a_*), which is the combination of the contributions of the three phases constituting soils: solid, water and air [9,13]. According to Corwin [14], three pathways of current flow contribute to the *EC_a_* measurement: current through the pore water solution (*the liquid phase pathway*); current through exchange complexes on the surface of soil colloids (*the soil-liquid phase pathway*); and current through the soil particles that are in direct contact (*the solid pathway*). *EC_a_* can be estimated from *ε_r_* readings [15] or from the electrical resistance that soil opposes to an alternating electric current [13,14]. *EC_p_* and *EC_a_* are strictly correlated, indeed an increase of ions in the matrix solution leads to an increase of *EC_a_* values [8,16,17].

Several models to estimate *EC_p_* from *EC_a_* have been developed in the last sixty years, based on empirical relations as well as on theoretical assumptions. Models are usually based on the empirical relationship between *EC_a_* and *θ* at constant *EC_p_* values, where the magnitude of *EC_a_* varies according to the tortuosity of the electrical current paths (depending on soil texture, density and particle geometry, particle pore distribution, and organic matter content). Tortuosity can be expressed in terms of a soil transmission factor (*π*) [16,18,19] or soil-type-related parameters [20–22].

Recent development of low-cost multi-sensor probes could make such *EC_p_* models implementable for continuous monitoring purposes. However, since most of the *EC_p_* models are calibrated in limited soil conditions [9,23–25], new relationships between variables and soil properties must be defined to extend their applicability to a wider range of soils.

The general aim of this study was to calibrate a multi-sensor probe for monitoring soil volumetric water content and soil water electrical conductivity in a heterogeneous saline coastal area. The specific objectives were: (*i*) to develop a procedure to simultaneously calibrate *θ* and *EC_p_*; (*ii*) to test different models for *EC_p_*; and (*iii*) to develop general functions to extend *EC_p_* model application to a wide range of soils, even in critical saline conditions.

## 3. Materials and Methods

### 3.1. Decagon ECH_2_O-5TE Probe

The sensor used in this experiment was an ECH_2_O-5TE probe (hereafter simply referred to as 5TE). 5TE is a multifunction sensor measuring *ε_r_*, *EC_a_*, and *T* (Decagon Devices Inc., Pullman, WA, USA). A detailed description of the 5TE can be found in Bogena *et al.*[26] and Campbell and Greenway [27]. The probe is a fork-type sensor (0.1 m in length, 0.032 m in height). Two of the three tines host the dielectric sensor. The capacitance sensor supplies a 70 MHz electromagnetic wave to the prongs that charge according to the dielectric of the soil surrounding the sensor. The reference soil volume is *ca.* 3 × 10^−4^·m^3^. A charge is consequently stored in the prongs and it is proportional to the soil dielectric. Previous versions of dielectric sensors by Decagon Devices operate at lower frequencies (e.g., ECHO10 probe, 5 MHz). The increase of operating frequency has led to a higher salinity tolerance [6,8,12]. In fact *ε_r_* measurement with 5TE should not be affected by soil salinity up to *EC_e_* values of 10 dS·m^−1^[28].

The bulk electrical conductivity is measured with a two-sensor array. The array consists of two screws placed on two of the sensor tines. An alternating electrical current is applied on the two screws and the resistance between them is measured. The sensor measures electrical conductivity up to 23.1 dS m^−1^ with 10% accuracy; however a user calibration is suggested above 7 dS·m^−1^. Temperature is measured with a surface-mounted thermistor reading the temperature on the surface of one of the prongs.

### 3.2. Soil Sampling

**Figure 1.**
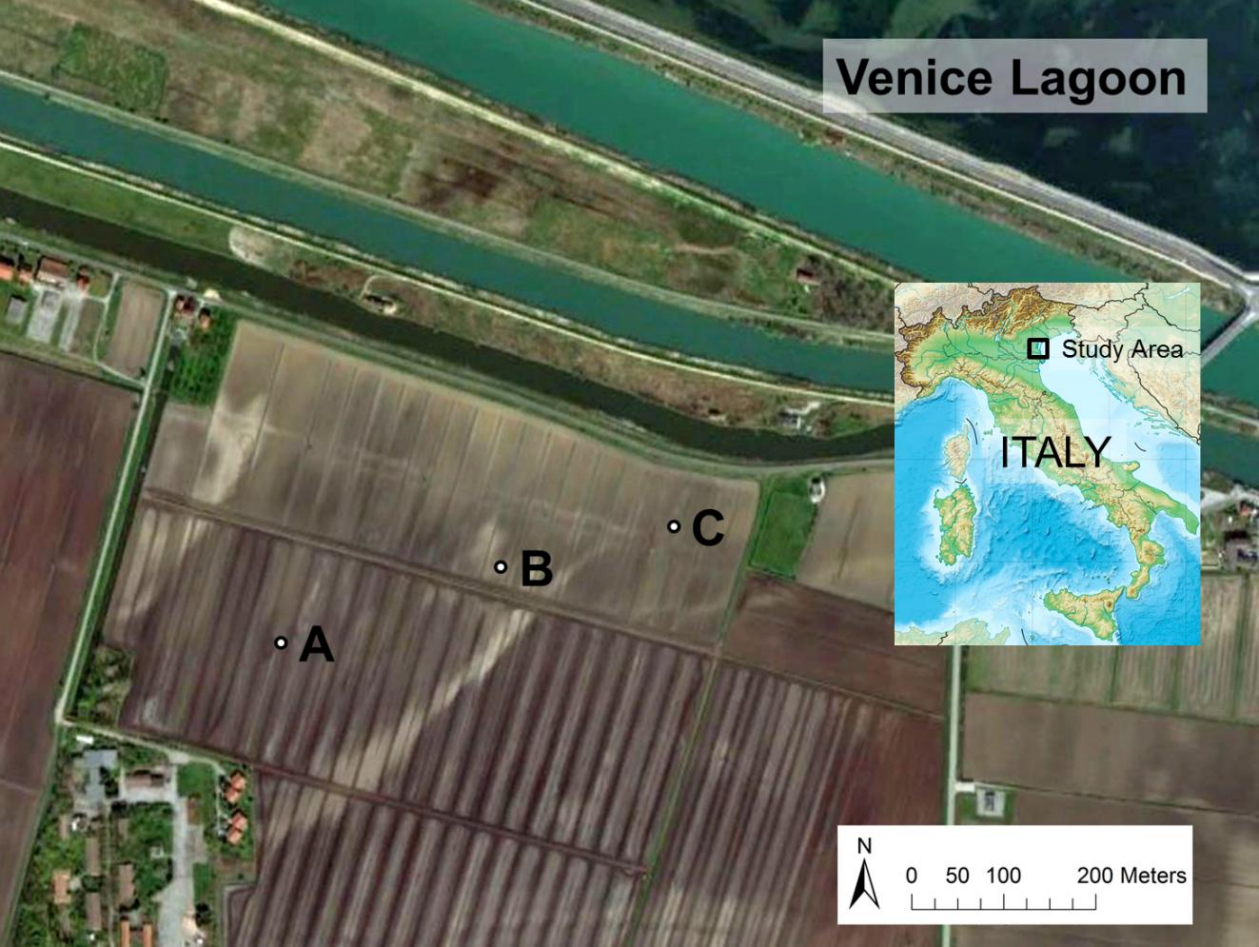
Aerial image of the study area at the southern edge of the Venice Lagoon, Italy. The sampling sites A, B, and C are marked.

Soil samples from a coastal farmland affected by saltwater intrusion [29,30] were cored for the calibration of the 5TE probe. The site is located at Ca’ Bianca, Chioggia (12°13′55.218″E; 45°10′57.862″N), just south of the Venice Lagoon, North-Eastern Italy. The area has high spatial variability in soil characteristics due to its deltaic origins (Figure 1).

Three sampling locations were chosen in the basin (sites A, B, and C, Figure 1). At sites A and B both topsoil (0 to 0.4 m depth) and subsoil (0.4 to 0.8 m depth) were collected, while only the topsoil was cored at site C since the profile is uniform. The main physical and chemical properties of the samples were characterized. Soil texture was determined with a laser particle size analyzer (Mastersizer 2000, Malvern Instruments Ltd., Great Malvern, UK). Soil total carbon content and soil organic carbon (SOC) content were analyzed with a Vario Macro Cube CNS analyzer (Elementar Analysensysteme GmbH, Hanau, Germany). Cation exchange capacity (CEC) was measured at a pH value of 8.2 according to the BaCl extraction method [31]. Soil pH was measured with a 1:2 soil to water ratio with a pH-meter (S47K, Mettler Toledo, Greifensee, Switzerland). Particle density (ρ_r_) was measured with an ethanol pycnometer [32]. Bulk density (ρ_b_) was determined from undisturbed core samples. *EC_e_* was measured according to Rhoades *et al.*[1].

Soil samples show high variability in sand (from 174.7 to 905.2 g·kg^−1^), organic carbon content (from 15.4 to 147.8 g·kg^−1^), and *EC_e_* values (from 0.61 to 6.38 dS·m^−1^). Five soil types were selected: a sandy soil with low SOC content and low *EC_e_*, a silty-clay-loam with low SOC content and high *EC_e_*, two loam and one clay-loam with medium-high SOC content. Main soil properties are listed in Table 1.

### 3.3. Experimental Settings

The 5TE probe was used in a mixture of soil (preliminarily air-dried and sifted at 2 mm) and saline solution (54.92% Cl^−^; 30.82% Na^+^; 7.68% SO_4_^2−^; 3.81% Mg^2+^; 1.21% Ca^2+^; 1.12% K^+^; 0.44%NaHCO_4_) to reproduce saline groundwater of the experimental site [33]. Soil samples were moistened to a relative saturation (*S*) of about 0, 0.35, 0.75, and 1.00 with a saline solution of 0, 5, 10, and 15 dS·m^−1^ (at 25 °C). The mixtures were prepared in a plastic container and then sealed and kept in a dark place at constant temperature 22 ± 1 °C for 48 hours. The soil was then packed uniformly in a 6 × 10^−4^·m^3^ beaker to reproduce the field bulk density. Output values for *ε_r_*, *EC_a_*, and *T* were recorded by a datalogger (Em50, Decagon Devices) connected to the 5TE probe.

Electrical conductivity of the wetting solution (*EC_w_*) differs from the electrical conductivity of the pore-water (*EC_p_*) [21]. Pore-water solution was extracted from a portion of the soil sample by vacuum displacement [34] at −90 kPa and *EC_p_* was measured with a S47K conductivity meter. *EC_e_* was then measured on the remaining soil sample. Water content was determined gravimetrically (at 105 °C for 24 hours). Measures were replicated 3 times.

### 3.4. Calibration Procedure

A three-step procedure was implemented to calibrate the sensor output for the collected samples: (1) model calibration to convert *ε_r_* and *EC_a_* readings to *θ* or *EC_p_*; (2) comparison and selection of the best models; (3) simultaneous calibration of the selected models for *θ* and *EC_p_* and evaluation of their robustness by applying a bootstrap procedure.

#### 3.4.1. Models to Convert *ε_r_* Readings to *θ*

Dielectric permittivity can be converted to volumetric water content using empirical models (e.g., [4]). However temperature and soil electrical conductivity affect the dielectric permittivity measurements of ECH_2_O sensors [5,35,36]. In one of their latest studies, Rosenbaum *et al.*[5] developed an empirical calibration to correct the temperature effect on *ε_r_* measurements which performed very well in both liquid and soil media. Investigating the effect of temperature on *ε_r_*, Bogena *et al*. [26] concluded that in a *T* range from 5 °C and 40 °C, *ε_r_* varies up to 8% with respect to the reference liquid used (*ε_r_* = 40 at 25 °C). As all the calibration experiments presented in this work took place at a controlled temperature of 22 ± 1 °C, the effect of *T* on *ε_r_* was considered negligible. On the other hand, *ε_r_* is much more sensitive to electrical conductivity changes [37].

Polynomial model-types as that proposed by Topp *et al*. [4] do not provide satisfactory *θ* estimates in the presence of high clay and organic contents or in saline soils, especially using sensors operating at low frequencies [12,38]. Indeed, application of the Topp model to the experimental data of Ca’ Bianca provided a large average error (∼0.11 m^3^·m^−3^).

Three models were tested to find a satisfactory empirical relationship between *ε_r_* and *θ* data for each soil at different *EC_w_* values, namely:

- logistic model:
  (2) $$\theta =\frac{\theta_{\mathrm{MAX}}}{1+e^{-\left(a+b\times \varepsilon_{r}\right)}}-U$$
- hyperbolic model:
  (3) $$\theta =\frac{\theta_{\mathrm{MAX}}\times a\times \varepsilon_{r}}{\theta_{\mathrm{MAX}}+a\times \varepsilon_{r}}$$
- logarithmic model:
  (4) $$\theta =a+b\times \text{ln}\left(\varepsilon_{r}\right)$$
  where *θ_MAX_* is the volumetric content at saturation, *a*, *b*, and *U* are fitting parameters.

The three models were compared with the Akaike Information Criterion (AIC) [39] and the one with the higher Akaike weight (W_AIC_) [40] was selected for the subsequent simultaneous calibration of *θ* and *EC_p_*. The Akaike Information Criterion (AIC) is a measure of the goodness of fit of a specific model. It allows the direct comparison of different concurrent equations for model selection purposes. AIC accounts for the risk of over-parameterization as well as for the goodness of fit; several models can be ranked according to their AIC, with the one having the lower value being the best. From the AIC, the Akaike weight (ΣW_AIC_ = 1) can be computed, which represents the probability that a specific model is the best, given the data and the set of candidate models. Note that the fitting parameters showed a high dependence on *EC_a_* and physico-chemical soil characteristics. To take this effect into account, the fitting parameters were expressed as a linear function of *EC_a_* and other selected soil properties yielding a “general” calibration equation usable on the various soils of the study site.

#### 3.4.2. Models to Convert *ε_r_* and *EC_a_* Readings to *EC_p_*

Four models were tested: the first is the Malicki and Walczak [21] model. They found that, when *ε_r_* is higher than 6.2, the slope ∂*EC_a_*/∂*ε_r_* depends only on salinity but not on water content, nor bulk density, nor dielectric permittivity. They developed an empirical relationship linearly linking *EC_a_* to *ε_r_* for various values of *EC_w_*, *i.e.*, *EC_a_*(*ε_r_*,*EC_w_*). The validity of the linear relationships holds above a “converging point” characterized by *ε_r0_* = 6.2 and *EC_a0_* = 0.08 dS·m^−1^. *EC_p_* was consequently defined as a function of *EC_a_*(*ε_r_*,*EC_w_*) and soil texture:

(5) $$EC_{p}=\frac{EC_{a}-EC_{a0}}{\left(\varepsilon_{r}-\varepsilon_{r0}\right)\times l}$$

where *l* is the slope of the relation between ∂*EC_a_*/∂*ε_r_* and *EC_w_*. This parameter depends on the sand content of the sample through the relation *l = l’+ l″* × *sand(%)*, with *l’* = 5.7 × 10^−3^ and *l″* = 7.1 × 10^−5^.

On the basis of Equation (5), Hilhorst [20] developed the following theoretical model:

(6) $$EC_{p}=\frac{\varepsilon_{p}\times EC_{a}}{\varepsilon_{r}-\varepsilon_{EC_{a}=0}}$$

where *ε_p_* is the real portion of the dielectric permittivity of the soil pore-water and *ε*_*EC*_*a*_=0_ is the real portion of the dielectric permittivity of the soil when bulk electrical conductivity is 0. *ε*_*EC*_*a*_=0_ is a soil-type dependent variable, even if Hilhorst recommended a value equal to 4.1 as a generic offset. Moreover, *ε_p_* was calculated as [20]:

(7) $$\varepsilon_{p}=80.3-0.37\times \left(T-20\right)$$

where *T* is the soil temperature in degrees Celsius, 80.3 is the real part of the complex permittivity of the pore-water at 20 °C, and 0.37 is a temperature correction factor. Hilhorst considers the imaginary part of *ε_r_* to be negligible, hence in his model *ε_r_* = *ε’*. The Hilhorst model was proved to perform correctly only for low *EC_p_* values. Hilhorst himself indicated an *EC_p_* value of 3 dS·m^−1^ as the upper limit for the validity of his model when a capacitance sensor operating at 30 MHz is used.

The third tested model is the one proposed by Rhoades *et al.*[16] (hereafter simply referred as Rhoades). They expressed the pore-water electrical conductivity as:

(8) $$EC_{p}=\frac{EC_{a}-EC_{s}}{\theta \times \pi }$$

where *EC_s_* (the electrical conductivity of the solid phase) was shown to be dependent on soil texture and through a linear correlation with clay content [24,41]; *π* is a tortuosity factor that mainly depends on soil hydraulic properties and was defined by Rhoades *et al.* as:

(9) π=c+d×θ

where the constants *c* and *d* can be estimated from the regression between *EC_a_* and *θ* at constant *EC_p_*[16].

Archie’s law [22] (hereafter simply referred as Archie) was developed to assess the conductivity of pore-water in clay-free rocks and sediments, and it has been therefore used in soils containing neither clay minerals nor organic matter. According to Archie *EC_p_* can be derived as follows:

(10) $$EC_{p}=k\times \frac{EC_{a}}{\phi^{m}\times S^{n}}$$

where *Φ* is the porosity (defined as *Φ* = 1 − *ρ_b_* × *ρ_r_*^−1^ = *θ_MAX_*), *S* the relative saturation (defined as *S = θ* × *Φ*^−1^), and *k*, *m* and *n* are fitting parameters. Allred *et al.*[13] showed that typical values of these three constants range from 0.5 to 2.5, from 1.3 to 2.5, and ∼2 for *k*, *m*, and *n*, respectively.

Archie has been modified in order to be used also in soils containing clay minerals [42] by simply considering the contribution of *EC_s_* in Equation (10). Hence, *EC_p_* was defined as:

(11) $$EC_{p}=k\times \frac{EC_{a}-EC_{s}}{\phi^{m}\times S^{n}}$$

Despite the fact that Archie was originally developed for deep sediments in oil research, it has been successfully applied in shallow groundwater systems to trace salinity. An example of such implementation is given by Monego *et al.*[43]. It is worth noticing that Archie and Rhoades show a similar formulation, being equal when *m* = 1 and *n* = 1 (then *k* = 1/π).

The four models apply for *θ* > 0.1 m^3^·m^−3^ (for Rhoades and Hilhorst), *θ* > 0.2 m^3^·m^−3^ (for Malicki and Walczak), and *S* > 0.3 (for Archie).

The models were tested with the experimental (*EC_a_*,*ε_r_*) values and the chemical and physical properties of the five soil samples collected at Ca’ Bianca. In a first step, the original formulations were tested by calculating the parameters according to the methodologies proposed by the authors. Next, the models were optimized by relating the calibration parameters to the physical and chemical characteristics of the soils. *EC_p_* data at *S* ≈ 0.35 were excluded from the optimization as it was impossible to collect a sufficient amount of solution with the extraction method used in this experiment. *EC_p_* data at *S* ≈ 0 were assumed equal to 0 dS·m^−1^[8].

#### 3.4.3. Simultaneous Calibration of Models for *θ* and *EC_p_*

The model parameters for the simultaneous quantification of *θ* and *EC_p_* were calibrated by minimizing the following objective function:

(12) $${RSS}_{\mathrm{tot}}=\sum_{i=1}^{N}{\left(EC_{p,i}-{\hat{EC}}_{p,i}\right)}^{2}+\sum_{j=1}^{M}{\left(W_{1}\times W_{2}\times \left(\theta_{j}-{\hat{\theta }}_{j}\right)\right)}^{2}$$

where *RSS_tot_* is the cumulative residual sum of squares, *M* and *N* are the total number of observed volumetric water content and pore-water electrical conductivity data, respectively, *EC_p,i_* and ${\hat{EC}}_{p,i}$, *θ_j_* and *θ̂_j_* are the observed and fitted *EC_p_* and *θ* values, respectively, *W_1_* and *W_2_* are two weighting factors. The parameter *W_1_* allows more weight to be given to one of the two variables. The parameter *W_2_* ensures that a proportional weight is given to the two residual sums of squares (*RSS*), and that the effect of having different units for *θ* and *EC_p_* is canceled. *W_2_* was calculated as suggested by Van Genuchten *et al.*[44]:

(13) $$W_{2}=\frac{M\times \sum_{i=1}^{N}EC_{p,i}}{N\times \sum_{j=1}^{M}\theta_{j}}$$

This weighted procedure prevents one data type (*i.e.*, *EC_p_* or *θ*) from dominating the other, solely because of its higher numerical values.

In this study the limited dataset size (*M* = 80 and *N* = 55) did not allow a validation to be performed on an independent set of data. The models were thus validated through a bootstrap procedure [45]. A *Y* number of iterations were carried out. At each iteration, a subset of 60 points out of 80 for *θ* and 42 out of 55 for *EC_p_* were extracted, forming the calibration dataset. The remaining points were retained for validation.

At the end of the iterations, the root mean square error ( $RMSE=\sqrt{\sum_{i=1}^{n}{\left(x_{i}-{\hat{x}}_{i}\right)}^{2}/n}$), which provides the goodness of fit, the median, and the 5th and 95th percentiles of the distribution of each parameter were retained for further analysis. The probability distribution function of RMSE was compared using the Kolmogorov-Smirnov (KS) test to assess the significance of difference in the model predictions.

The calibration procedure described above was performed using the Generalized Reduced Gradient (GRG) Nonlinear Solving Method (Frontline Systems, Inc., Incline Village, NV, USA).

## 4. Results and Discussion

### 4.1. Converting ε_r_ Readings to θ

**Figure 2.**
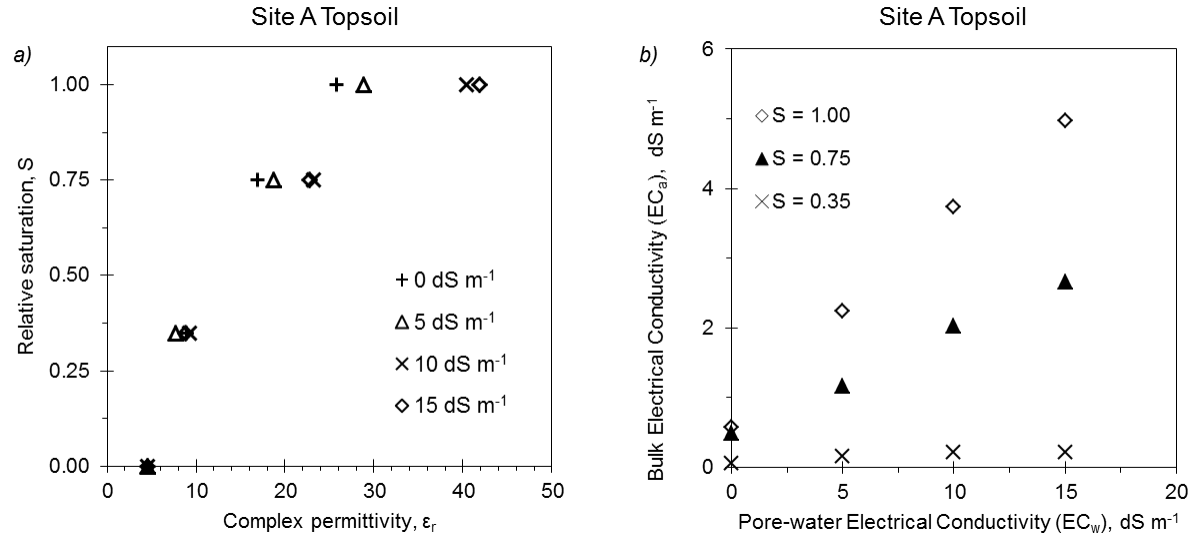
Site A, topsoil: (**a**) relative saturation vs. measured complex permittivity for four *EC_w_* values of the wetting solution; (**b**) influence of *EC_w_* on bulk electrical conductivity at various relative saturation levels.

Dependence of 5TE on bulk electrical conductivity was observed to be similar in all the tested soil samples. *ε_r_* readings were greatly affected by *EC_a_*: especially for high *θ* values, a small increase in *EC_w_* significantly raised the dielectric output of the probe, indicating that dielectric readings carried out in highly conductive media must be corrected. This finding confirms the results by Rosenbaum *et al.*[5] on the same probe and by Saito *et al.*[8] on other Decagon dielectric probes operating at lower frequencies. An example of the non-linear response of *ε_r_* at different *EC_w_* and *θ* values is presented in Figure 2(a). Starting from a relative saturation of 0.75, the response of the probe significantly diverged at salinity solution with *EC_w_* > 10 dS m^−1^. Figure 2(b) evidences also the direct effect of the *EC_w_* on *EC_a_* readings and how the effect was amplified at higher water content. This observation, confirmed by Schwank *et al.*[11] and Rosenbaum *et al.*[5], suggests investigating the effect of *EC_a_* on *θ* estimation.

Between the tested *θ* models, Equation (4) showed the best performances, with an Akaike weight *W_AIC_* close to 1 (Table 2).

Parameters *a* and *b* of the logarithmic model were found to be significantly correlated with the *EC_a_* values at different water contents. Therefore the logarithmic model was reformulated as:

(14) $$\theta =\left(a^{\prime }+a^{\prime \prime }\times EC_{a}\right)+\left(b^{\prime }+b^{\prime \prime }\times EC_{a}\right)\times \text{ln}\left(\varepsilon_{r}\right)$$

where *a′*, *a″*, *b’*, and *b″* are empirical parameters. Calibration of Equation (14) highlighted a strong correlation between the terms *a′* × *EC_a_*+ *a″* and *b’*× *EC_a_*+ *b″*. Consequently this latter term was assumed equal to *q*× (*a′×EC_a_*+ *a″*), where *q* is a proportionality constant. Equation (14) could thus be reformulated as:

(15) $$\theta =\left(a^{\prime }+a^{\prime \prime }\times EC_{a}\right)\times \left(1+q\times \text{ln}\left(\varepsilon_{r}\right)\right)$$

Equations (14) and (15) were compared with the AIC test. A W_AIC_ = 0.99 was obtained for Equation (15), indicating that this formulation of the logarithmic model is to be preferred over Equation (14), mainly for the reduced number of parameters.

To identify a “general” equation, *q* was set as a constant (=−0.766), whereas parameters *a′* and *a″* were related to soil properties (Table 3). Parameters *a′* and a″ were estimated according to the following empirical equations:

(16) $$a^{\prime }=-0.352-0.006\times SOC\left(\%\right)$$

(17) $$a^{\prime \prime }=0.020-0.009\times \frac{\mathrm{clay}\left(\%\right)}{\mathrm{sand}\left(\%\right)}$$

Equation (15) allows correcting of the effect of dielectric losses due to the high electrical conductivity of the medium [3] due to high organic carbon content, salinity, and clay/sand ratio. The RMSE of Equation (15) was 0.038 m^3^·m^−3^.

### 4.2. Converting ε_r_ and EC_a_ Readings to EC_p_

The parameters of models (5), (6), (8), and (11) showed significant correlations with soil properties (Table 3).

The parameter *l* by Malicki and Walczak was confirmed to be mainly correlated to sand content. The calibrated parameters for Equation (5) are: *EC_a0_* = 0.06 dS·m^−1^; *ε_r0_* = 7.1; *l =* 0.012 + 10^−6^*× sand(%)* yielding RMSE = 2.52 dS·m^−1^. In the original paper by Malicki and Walczak *l* varied from 0.0083 to 0.0127 while in this experiment the range was narrower, from 0.0117 to 0.0124.

The *ε_(ECa = 0)_* parameter by Hilhorst was expressed as a function of soil organic carbon content:

(18) $$\varepsilon_{EC=0}=4.851+0.203\times SOC\left(\%\right)$$

According to Equation (18), *ε_ECa = 0_* ranged from 5.16 to 7.85, values close to the interval found by Hilhorst (from 3.76 to 7.6 in soils and synthetic media). The calibrated model yielded RMSE = 2.34 dS m^−1^.

Concerning the Rhoades and Archie models, the term *EC_s_* was neglected as the 5TE probe registered *EC_a_* = 0 in dry soil conditions. Please note that other Authors [41] demonstrated that EC_s_ could assume a certain magnitude.

In contrast to Equation (9) by Rhoades *et al.*[16], *π* was found to be uncorrelated with soil water content. Nevertheless, *π* showed a linear correlation with soil porosity (*π = e + f × ϕ*), with *e* and *f* depending on CaCO_3_ as follows:

(19) $$\pi =\left(0.129\times {\mathrm{CaCO}}_{3}\left(\%\right)-1.779\right)+\left(-0.232\times {\mathrm{CaCO}}_{3}\left(\%\right)+3.989\right)\times \phi$$

In the tested samples *π* ranged from 0.22 to 0.71, whereas Rhoades *et al.*[16] found a variation from 0.01 to 0.6. The inverse correlation of CaCO_3_ with the tortuosity factor evidenced in the Ca’ Bianca soils can be explained by the fact that here a low CaCO_3_ content corresponds to high clay and SOC percentages. Indeed, the higher clay and SOC contents (more complicated geometric arrangement), the higher is soil tortuosity [41]. The “general” formulation of the Rhoades model provided RMSE = 0.90 dS m^−1^.

Several formulations were attempted for Archie in order to decrease the number of parameters related to soil properties. Here, the parameters *k*, *m*, and *n* were alternately fixed and kept independent from the soil type. The formulation with *k* = 0.487 provided the best fitting according to the AIC test. With fixed *k*, *n* showed a significant correlation with sand content:

(20) n=−0.669+0.035×sand(%)

**Figure 3.**
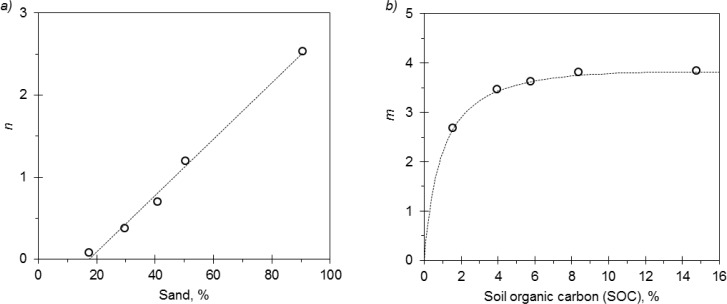
Archie model: relationships (**a**) *n vs.* sand content and (**b**) *m vs.* soil organic carbon. The dotted line represents the fit described by Equations (20) and (21), respectively. For the latter, RSS = 2.46 × 10^−3^ and RMSE = 0.04.

It is worth noticing that with higher sand contents (Figure 3(a)) *n* ≅ 2.5, which is close to *n* values suggested for sandy media [13]. As shown in Table 3, *n* decreases with increasing clay values. For given *S* and *EC_a_* values, it is clearly derived from Equation (10) that the smaller the *n* the higher is *EC_p_*, *i.e.*, with a large percentage of clay the influence of “*the liquid phase pathway*” on the *EC_a_* reading is reduced [14,41]. A non-linear relationship was detected between *m* and soil organic carbon (Figure 3(b)):

(21) $$m=-0.018\times SOC\left(\%\right)+\frac{4.350\times SOC\left(\%\right)}{SOC\left(\%\right)+0.966}$$

Values of *m* between 2.65 and 3.82 were derived. As reported by Archie, *m* becomes larger as the permeability of the porous medium decreases (increasing tortuosity). As shown in Figure 3(b) the magnitude of *m* rises with SOC. High organic contents decrease soil bulk density, possibly increasing soil tortuosity [46]. Archie calibration returned RMSE = 0.65 dS m^−1^.

Comparison between the RMSE values computed for the four *EC_p_* models showed that the “general” formulation of Archie provided the best estimates. Archie also had the highest *W_AIC_* (∼1.00). For the Malicki and Walczak, Hilhorst, and Rhoades models the *W_AIC_* were close to zero.

**Figure 4.**
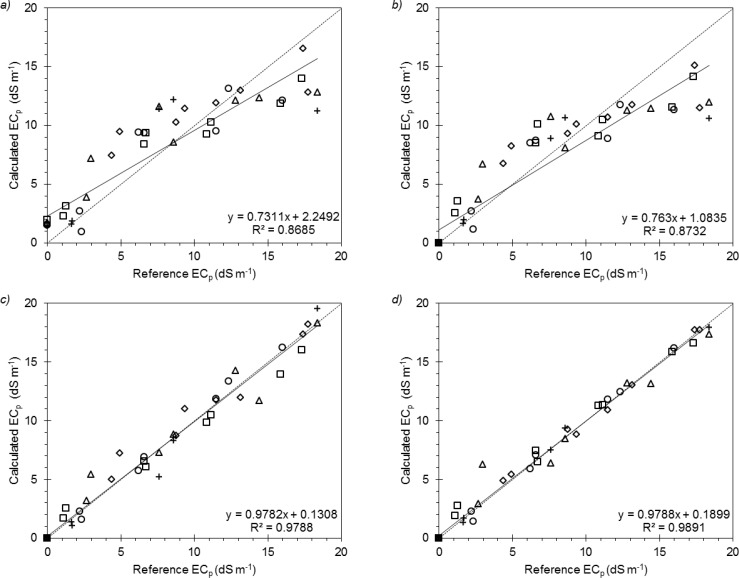
Comparison of calculated vs. reference pore-water electrical conductivity for the five soil samples using the “general” (**a**) Malicki and Walczak, (**b**) Hilhorst, (**c**) Rhoades, and (**d**) Archie models.; The symbols refer to: □ site A, topsoil; ♦ site A, subsoil; ○ site B, topsoil; + site B, subsoil; and Δ site C, topsoil.

These results are also confirmed by the linear regressions between measured and estimated *EC_p_* (Figure 4). As displayed in this figure the models by Malicki and Walczak, and by Hilhorst did not show a good fitting, especially at high *EC_p_* values, as already observed by [23,25].

The different performances of the four models at various salinity ranges were tested resampling observed and estimated *EC_p_* 2,000 times, to compute average RMSEs and their confidence intervals at *p* = 0.05 as previously done by Giardini *et al.*[47]. The selected ranges were: (a) the 0–3 dS·m^−1^ and >3 dS·m^−1^; and (b) the 0–10 dS·m^−1^ and >10 dS·m^−1^.

At low *EC_p_* range (*i.e.*, *EC_p_* < 3 dS·m^−1^) Rhoades showed the smallest RMSE (0.57 dS·m^−1^), nevertheless its performance was not significantly different from those by Hilhorst (RMSE = 0.93 dS·m^−1^) and Archie (RMSE = 0.72 dS·m^−1^). On the contrary, the model by Malicki and Walczak provided significantly higher errors (RMSE = 1.69 dS·m^−1^).

Above 3 dS·m^−1^, the models by Malicki and Walczak and by Hilhorst significantly differentiated from the other two. In fact they generally overestimated *EC_p_* in the range from 3 to 10 dS·m^−1^ with RMSE equal to 2.16 and 1.43 dS·m^−1^, respectively. On the other hand they underestimated *EC_p_* when the pore-water was very conductive (*i.e.*, *EC_p_* > 10 dS·m^−1^), with RMSE = 3.28 dS·m^−1^ and RMSE = 3.83 dS·m^−1^, respectively.

In their work, Malicki and Walczak used TDR probes at fairly high frequencies, reducing the influence of *EC_a_* on *ε_r_*. Moreover, their study was conducted using a wetting solution with a maximum conductivity of 11.7 dS·m^−1^. In the present work, calibrating the Malicki and Walczak model only for *EC_p_* < 10 dS·m^−1^ would provide satisfactory estimations (RMSE = 1.00 dS·m^−1^). Moreover, the metrics of fitting regression would have shown a slope and intercept of 0.837 and 0.680, yielding very similar results to those obtained by Malicki and Walczak in their work. With some limitations, the model by Malicki and Walczak might therefore be used in capacitance applications as well as TDR [21] and frequency-domain reflectometry [10].

Hilhorst validated his model in a much lower *EC_p_* range than the one used in this work. Hilhorst actually indicated the validity upper bound for the probe used in his work as 3 dS·m^−1^. Indeed, in the present study the model showed good performances in the 0–3 dS·m^−1^ range. Moreover, calibrating the model for *EC_p_* <10 dS·m^−1^ would suitably yield a RMSE of 0.68 dS·m^−1^ with an observed-estimated relationship having a slope and an intercept of 0.957 and 0.127, respectively. Most likely, the higher operating frequency of 5TE compared to the capacitive probe used by Hilhorst (*i.e.*, 30 MHz) could have increased the range of model validity. However, as stated by Hilhorst, the model assumptions cease to be valid at higher salt concentrations as *ε_p_* significantly deviates from that of free water (Equation (7)). From the experiment presented here this limit seems to be *EC_p_* ∼ 10 dS·m^−1^.

The comparison of the error distribution at different *EC_p_* ranges showed that Rhoades and Archie did not give significantly different performances. Nevertheless, the Rhoades model showed a larger RMSE at high *EC_p_* values than at low ones (*EC_p_* < 10 dS·m^−1^: RMSE = 0.78 dS·m^−1^; *EC_p_* > 10 dS·m^−1^: RMSE = 1.17 dS·m^−1^). On the other hand, the Archie model showed a greater consistency over the two salinity ranges (*EC_p_* < 10 dS·m^−1^: RMSE = 0.69 dS·m^−1^; EC_p_ > 10 dS·m^−1^: RMSE = 0.54 dS·m^−1^).

### 4.3. Simultaneous Calibration of Models for θ and EC_p_

As reported above, the “general” formulations of Rhoades and Archie showed overall similar performances. As already stated experimental *θ* values were used in the two equations. A simultaneous calibration was then done estimating *EC_p_* and *θ* from *EC_a_* and *ε_r_* readings by substituting the “general” logarithmic *θ* model (Equation (15)) within Rhoades and Archie “general” models. The *W_1_* weight (Equation (12)) was set to 0.5, thus improving the *EC_p_* estimation without notably worsening the *θ* evaluation.

The combined logarithmic *θ* model and Rhoades reads:

(22) $$EC_{p}=\frac{EC_{a}}{\left({a^{\prime }}_{\mathrm{Rhoades}}+{a^{\prime \prime }}_{\mathrm{Rhoades}}\times EC_{a}\right)\times \left(1-0.742\times \text{ln}\left(\varepsilon_{r}\right)\right)\times \pi_{\mathrm{Rhoades}}}$$

with:

(23) $${a^{\prime }}_{\mathrm{Rhoades}}=-0.427-4.0\cdot 10^{-5}\times SOC\left(\%\right)$$

(24) $${a^{\prime \prime }}_{\mathrm{Rhoades}}=0.024-0.008\times \frac{\mathrm{clay}\left(\%\right)}{\mathrm{sand}\left(\%\right)}$$

(25) $$\pi_{\mathrm{Rhoades}}=\left(0.074\times {\mathrm{CaCO}}_{3}\left(\%\right)-1.354\right)+\left(-0.132\times {\mathrm{CaCO}}_{3}\left(\%\right)+3.232\right)\times \phi$$

where *a′_Rhoades_*, *a″_Rhoades_*, and *π_Rhoades_* are the fitting parameters defined in Equations (16), (17), and (19) during the independent calibration of *θ* and *EC_p_*.

Similarly, the combined logarithmic *θ* model and Archie becomes:

(26) $${EC}_{p}=0.466\times \frac{{EC}_{a}}{\phi^{m_{\mathrm{Archie}}}{\left(\frac{\left({a^{\prime }}_{\mathrm{Archie}}+{a^{\prime \prime }}_{\mathrm{Archie}}\times {EC}_{a}\right)\times \left(1-0.738\times \text{ln}\left(\varepsilon_{r}\right)\right)}{\phi }\right)}^{n_{\mathrm{Archie}}}}$$

with:

(27) $${a^{\prime }}_{\mathrm{Archie}}=-0.427-0.006\times SOC\left(\%\right)$$

(28) $${a^{\prime \prime }}_{\mathrm{Archie}}=0.036-0.012\times \frac{\mathrm{clay}\left(\%\right)}{\mathrm{sand}\left(\%\right)}$$

(29) $$m_{\mathrm{Archie}}=-2.8\cdot 10^{-4}\times SOC\left(\%\right)+\frac{4.134\times SOC\left(\%\right)}{SOC\left(\%\right)+0.719}$$

(30) n=−0.697+0.040×sand(%)

where *a′_Archie_*, *a″_Archie_*, *m_Archie_*, and *n_Archie_* are the fitting parameters originally defined in Equations (16), (17), (21), and (20).

The calibration of Equation (22) yielded RSME values for *θ* and *EC_p_* of 0.048 m^3^·m^−3^ and 0.77 dS·m^−1^, respectively. Better overall results were obtained by Equation (26): RMSE = 0.046 m^3^·m^−3^ and RMSE = 0.63 dS·m^−1^ for *θ* and *EC_p_*, respectively. It is worth noting that the simultaneously calibrated parameters were very close to the independently calibrated ones.

A bootstrap validation was done on the simultaneous calibrations. A total of 5,000 iterations were operated for both Equations (22) and (26). Table 4 shows the variations of the slope and intercept of the fitting linear regression between observed and predicted values. Soil water content was correctly predicted by both the equations: the slope and intercept medians of the observed-estimated relationships were fairly close to 1 and 0, respectively. *EC_p_* predictions were less accurate, generally overestimated by Equation (22) and underestimated by Equation (26) (Table 4).

**Figure 5.**
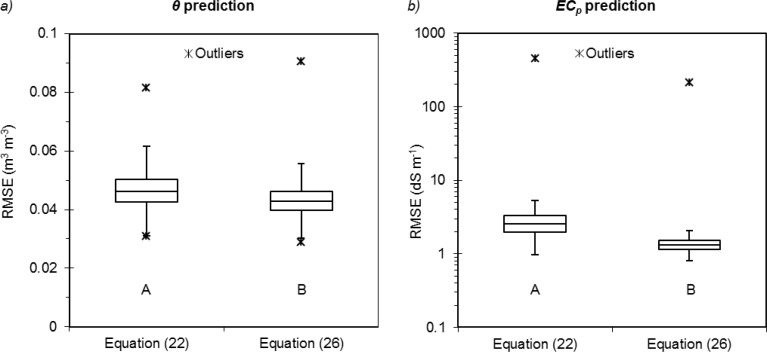
Comparison between the prediction performance of Equations (22) and (26) according to the Kolmogorov-Smirnov test. Boxplot for the RMSE values of (**a**) volumetric water content and (**b**) pore-water electrical conductivity. The letters A and B in the boxes indicate a significant difference (*p* < 0.01) between the RMSE distributions.

According to the Kolmogorov-Smirnov test, significant differences were observed between the two equations. The Archie-based model provided significantly lower RMSE values on the validation sets for both *θ* (*p* < 0.01) and *EC_p_* (*p* < 0.01) (Figure 5(a,b)). Equations (22) or (26) provided similar maximum errors for water content, with maximum RMSE of 0.08 m^3^·m^−3^ and 0.09 m^3^·m^−3^, respectively. On the other hand, Equation (22) produced a maximum *EC_p_* error higher than that of Equation (26) (451.42 dS·m^−1^*vs.* 211.26 dS·m^−1^). The overall more accurate prediction of the system implementing Archie can be justified by the more flexible functional form of the *EC_p_* model allowed by the two exponential parameters.

## 5. Summary and Conclusions

Low-cost capacitance-resistance multiprobe sensors are becoming popular for agro-environmental studies. In order to obtain reliable results, robust models for soil water content and pore-water electrical conductivity must be calibrated in different soil and climatic conditions, especially when these instruments are used in coastal areas with contrasting soils and affected by saltwater contamination.

This experiment verifies the possibility of simultaneously quantifying water content and pore-water electrical conductivity from complex permittivity, bulk electrical conductivity, and soil temperature measurements performed by the ECH_2_O-5TE (Decagon Devices, Inc.) probe. This result was achieved by improving empirical/theoretical reference models with the use of parameters dependent on physical and chemical soil properties, such as texture, soil organic carbon and soil carbonates. The improved models, in particular the one developed starting from Archie’s law, prove to be reliable and robust over a wide range of water content (from dry to saturated conditions), salinity conditions (pore-water electrical conductivity from 0 to ∼20 dS·m^−1^), and soil types (from sand with low SOC to clay-loam with high SOC).

Further studies performed in different soil and climatic environment coupled with improved dielectric sensors (e.g., with higher operating frequencies) will allow the accuracy of soil water content and pore-water salinity determination to be increased.

## Figures and Tables

**Table 1.** Texture, total and organic carbon content, cation exchange capacity, pH, particle density, bulk density, and conductivity of the saturated paste extract for the five soil samples collected in the Ca’ Bianca sites and used in this study.

**Soil Sample**		**Sand (%)**	**Silt (%)**	**Clay (%)**	**Total C (%)**	**SOC (%)**	**CEC (meq·g^−1^)**	**pH**	**ρ_r_ (g**·**cm^−3^)**	**ρ_b_ (g**·**cm^−3^)**	**EC_e_ (dS·m^−1^)**

**A**	**Topsoil**	40.92	41.31	17.77	15.50	14.78	0.57	5.60	1.90	0.87	0.61
**A**	**Subsoil**	17.47	52.66	29.87	4.30	3.96	0.12	5.89	2.28	1.08	6.38
**B**	**Topsoil**	50.54	37.61	11.85	6.64	5.78	0.33	7.23	2.32	1.07	1.42
**B**	**Subsoil**	90.52	7.71	1.77	4.26	1.54	0.05	7.68	2.62	1.29	2.26
**C**	**Topsoil**	29.61	48.46	21.93	9.84	8.36	0.45	7.58	2.21	0.93	2.05

**Table 2.** Outcome of the *θ* model comparison according to Akaike information criterion [40]: residual mean squares (RMS), total number of parameters (K—number of parameters of the model including the variance of the estimated residuals), Akaike Information Criterion (AIC), AIC differences (D_i_), and Akaike weights (*W_AIC_*).

**Model**	**RMS**	**K**	**AIC**	**D_i_**	**W_AIC_**
**Hyperbolic**	0.015	21	−301.61	104	2.11 × 10^−23^
**Logistic**	0.002	42	−391.41	15	6.69 × 10^−4^
**Logarithmic**	0.001	41	−406.03	0	1.000

**Table 3.** Pearson linear correlation coefficients for some soil properties and the parameters in Equations (15) (logarithmic model), (5) (Malicki and Walczak), (6) (Hilhorst), (19) (Rhoades tortuosity) and (10) (Archie). Bold numbers indicate a significant linear relationship.

	**Equation (15)**		**Equation (5)**	**Equation (6)**	**Equation (19)**		**Equation (10)**	
	***a′***	***a″***	***l***	**ε***_ECa = 0_*	***e***	***f***	***m***	***n***
**Sand**	*0.25*	*0.76*	**1.00**	−*0.60*	−*0.68*	*0.73*	−*0.79*	**1.00**
**Clay**	−*0.14*	−*0.8*	−**0.98**	*0.51*	*0.59*	−*0.62*	*0.66*	−**0.97**
**Clay/Sand**	*0.23*	−**0.89**	−*0.82*	*0.20*	*0.45*	−*0.43*	*0.31*	−*0.79*
**SOC**	−**0.98**	*0.30*	−*0.40*	**0.94**	*0.36*	−*0.48*	*0.78*	−*0.46*
**CaCO_3_**	*0.26*	*0.58*	*0.85*	−*0.73*	−**0.95**	**0.96**	−*0.75*	*0.84*

**Table 4.** Statistical analysis of the bootstrap validation outcome: median, 5th, and 95th percentile of slope and intercept distributions of the observed-predicted relationships for volumetric water content and pore-water electrical conductivity using Equations (22) and (26).

	**Slope**			**Intercept**		
	**Median**	**5% Limit**	**95% Limit**	**Median**	**5% Limit**	**95% Limit**
***θ***						
**Rhoades (Equation (22))**	0.97	0.89	1.02	0.01	−0.01	0.04
**Archie (Equation (26))**	0.98	0.91	1.04	0.01	−0.01	0.03

***EC****_p_*						
**Rhoades (Equation (22))**	1.15	0.98	1.31	0.13	−0.13	0.39
**Archie (Equation (26))**	0.93	0.88	1.03	0.32	0.11	0.53

## Symbols

*θ*: volumetric water content
*ε_r_*: soil complex permittivity
*EC_a_*: bulk electrical conductivity
*EC_p_*: pore-water electrical conductivity
*EC_w_*: electrical conductivity of the solution used to wet the soil
*EC_s_*: electrical conductivity of the solid phase
*EC_e_*: electrical conductivity of aqueous extract of saturated soil-paste
